# The iron–sulfur cluster biosynthesis protein SUFB is required for chlorophyll synthesis, but not phytochrome signaling

**DOI:** 10.1111/tpj.13455

**Published:** 2017-02-08

**Authors:** Xueyun Hu, Mike T. Page, Akihiro Sumida, Ayumi Tanaka, Matthew J. Terry, Ryouichi Tanaka

**Affiliations:** ^1^ Institute of Low Temperature ScienceHokkaido UniversitySapporo060‐0819Japan; ^2^ School of Life Science and EngineeringSouthwest University of Science and TechnologyMianyang621010China; ^3^ Biological SciencesUniversity of SouthamptonSouthamptonUK; ^4^ Institute for Life SciencesUniversity of SouthamptonSouthamptonUK

**Keywords:** chloroplast, Fe–S cluster, chlorophyll biosynthesis, Mg‐protoporphyrin monomethyl ester cyclase, far‐red light, *Arabidopsis thaliana*

## Abstract

Proteins that contain iron–sulfur (Fe–S) clusters play pivotal roles in various metabolic processes such as photosynthesis and redox metabolism. Among the proteins involved in the biosynthesis of Fe–S clusters in plants, the SUFB subunit of the SUFBCD complex appears to be unique because SUFB has been reported to be involved in chlorophyll metabolism and phytochrome‐mediated signaling. To gain insights into the function of the SUFB protein, we analyzed the phenotypes of two *SUFB* mutants, *laf6* and *hmc1*, and RNA interference (RNAi) lines with reduced *SUFB* expression. When grown in the light, the *laf6* and *hmc1* mutants and the *SUFB* RNAi lines accumulated higher levels of the chlorophyll biosynthesis intermediate Mg‐protoporphyrin IX monomethylester (Mg‐proto MME), consistent with the impairment of Mg‐proto MME cyclase activity. Both SUFC‐ and SUFD‐deficient RNAi lines accumulated the same intermediate, suggesting that inhibition of Fe‐S cluster synthesis is the primary cause of this impairment. Dark‐grown *laf6* seedlings also showed an increase in protoporphyrin IX (Proto IX), Mg‐proto, Mg‐proto MME and 3,8‐divinyl protochlorophyllide *a* (DV‐Pchlide) levels, but this was not observed in *hmc1* or the *SUFB* RNAi lines, nor was it complemented by *SUFB* overexpression. In addition, the long hypocotyl in far‐red light phenotype of the *laf6* mutant could not be rescued by *SUFB* overexpression and segregated from the pale‐green SUFB‐deficient phenotype, indicating it is not caused by mutation at the *SUFB* locus. These results demonstrate that biosynthesis of Fe–S clusters is important for chlorophyll biosynthesis, but that the *laf6* phenotype is not due to a *SUFB* mutation.

## Significance Statement

We demonstrate that Fe‐S biosynthesis is required for Mg‐protoporphyrin monomethylester cyclase activity, an essential step in the synthesis of the chlorophyll precursor protochlorophyllide. However, we show that the long hypocotyl phenotype reported for the *laf6* mutant is not due to a deficiency in the Fe‐S biosynthesis protein SUFB, but instead is the result of a second site mutation.

## Introduction

Iron–sulfur (Fe–S) cluster cofactors play pivotal roles in various biological processes that are critical to life, such as photosynthesis and redox metabolism (Sheftel *et al*., 2010; Balk and Pilon, 2011). In plastids, Fe–S proteins are involved in photosynthetic electron transport and nitrogen and sulfur assimilation; therefore Fe–S cluster biogenesis is essential for these processes. Several proteins have been shown to take part in the biogenesis of Fe–S clusters in plastids, with the sulfur mobilization (SUF) system suggested to play a central role (Balk and Pilon, 2011). In an accompanying paper (Hu *et al*., 2017) we provided *in planta* evidence that demonstrates the importance of the SUF system in general biosynthesis of Fe–S clusters in plastids.

In the SUF system, the SUFB, SUFC and SUFD proteins form a complex, which is suggested to act as a scaffold to form Fe–S clusters. The Fe–S clusters are subsequently transferred to specific Fe–S carrier proteins before they are incorporated into Fe–S apoproteins (Balk and Pilon, 2011; Couturier *et al*., 2013; Balk and Schaedler, 2014). This model is based on the mechanism revealed for the bacterial SUF proteins. In *Escherichia coli*, one SUFB, two SUFC and one SUFD protein form a SUFBC_2_D complex that has been shown to function as a scaffold for Fe–S cluster assembly *in vitro* (Outten *et al*., 2003; Chahal *et al*., 2009; Wollers *et al*., 2010). The protein sequences of plant SUFBs exhibit high similarity to their prokaryotic counterparts, and Arabidopsis SUFB can complement SUFB deficiency in *E. coli* (Xu *et al*., 2005). In addition, SUFB has been shown to interact with the evolutionarily conserved plastidic SUFC protein (Xu *et al*., 2005), which in turn can interact with Arabidopsis plastidic SUFD (Xu and Møller, 2004). Recently, we demonstrated that Arabidopsis SUFB, SUFC and SUFD form a protein complex similar to their bacterial counterparts, indicating that the plant SUFBCD complex also functions as a scaffold for Fe–S cluster biosynthesis (Hu *et al*., 2017). For better understanding of the roles of Fe–S cluster biosynthesis in plastids, various mutant plants in which Fe–S synthesizing activities are compromised have been analyzed. These mutants typically show a reduction in photosystem I (PSI) subunits that bind several Fe–S proteins, as well as reduced levels of chlorophyll and a loss of accumulation of many photosynthetic proteins, including those without Fe–S clusters (Lezhneva *et al*., 2004; Touraine *et al*., 2004; Yabe *et al*., 2004; Van Hoewyk *et al*., 2007). However, the underlying reason for the reduced chlorophyll phenotypes is not currently established.

In higher plants, chlorophyll is synthesized from glutamate (Tanaka and Tanaka, 2007; Mochizuki *et al*., 2010; see Figure S1 in the Supporting Information). Three enzymatic steps synthesize 5‐aminolevulinic acid (ALA) from glutamate and eight molecules of ALA are subsequently converted into protoporphyrinogen IX. Protoporphyrinogen IX is then oxidized by protoporphyrinogen IX oxidase to protoporphyrin IX (Proto IX), which is converted into either Mg‐protoporphyrin IX (Mg‐proto) by Mg‐chelatase or protoheme by ferrochelatase. This is the main branching point of chlorophyll biosynthesis. Mg‐proto is further esterified to form Mg‐proto monomethyl ester (Mg‐proto MME). Conversion of Mg‐proto MME to divinyl protochlorophyllide *a* (DV‐Pchlide) by Mg‐proto MME cyclase follows, and DV‐Pchlide is then modified to form monovinyl protochlorophyllide *a* (MV‐Pchlide). Upon illumination, MV‐Pchlide is reduced to chlorophyllide *a*, which is then esterified to form chlorophyll.

A number of Arabidopsis *SUF* mutants have been characterized to date and all show a pale green phenotype. This includes the *SUFB* mutant allele, *long after far‐red 6* (*laf6*)*,* which harbors a transposon insertion in the upstream untranslated region of the *SUFB* gene resulting in reduced *SUFB* expression (Møller *et al*., 2001), and *hmc1* that has a base substitution in its coding sequence resulting in the replacement of a conserved proline with a leucine (Nagane *et al*., 2010). Similarly, a *SUFD* T‐DNA insertion mutant of Arabidopsis also showed a pale green phenotype with a reduced chlorophyll content (Hjorth *et al*., 2005). These observations demonstrate that chlorophyll accumulation requires Fe–S cluster synthesis in Arabidopsis (Nagane *et al*., 2010; Balk and Schaedler, 2014). In addition to reduced chlorophyll accumulation, the two *SUFB* alleles mentioned above were both reported to accumulate intermediates of chlorophyll metabolism: Proto IX for *laf6* (Møller *et al*., 2001) and 7‐hydroxymethyl‐chlorophyll *a* and pheophorbide *a*, both of which are intermediates of chlorophyll breakdown, for *hmc1* (Nagane *et al*., 2010). Since there has been no report of mutants in other SUF proteins accumulating intermediates of chlorophyll metabolism, it was speculated that SUFB might play a specific role in the regulation of chlorophyll metabolism in addition to functioning in the biosynthesis of Fe–S clusters (Nagane *et al*., 2010). SUFB has also been proposed to be involved in phytochrome signaling. The *laf6* mutant exhibited long hypocotyl when seedlings were grown in far‐red (FR) light, suggesting that the *laf6* mutation affects phytochrome A signaling (Møller *et al*., 2001). However, long hypocotyl phenotype has not been reported for the other two *SUF* mutants and the role of SUFB in phytochrome signaling remains unclear.

Since it was reported that a knockout of *SUFB* was lethal at the embryonic stage (Nagane *et al*., 2010), we have investigated the physiological roles of SUFB and Fe–S cluster biosynthesis by constructing conditional *SUFB* silencing lines using RNA interference (RNAi) technology. In addition, we have expressed *SUFB* in the *laf6* mutant background to evaluate the effects of *SUFB* overexpression on its hypocotyl elongation phenotype. Through in‐depth analysis of these lines, we have been able to identify potential target sites of Fe‐S cluster biosynthesis deficiency within the chlorophyll biosynthesis pathway and have clarified the hypotheses about the specific functions of SUFB in chlorophyll biosynthesis and phytochrome signaling.

## Results

### Depletion of SUFB by RNAi results in reduced chlorophyll levels

**Figure 1:**
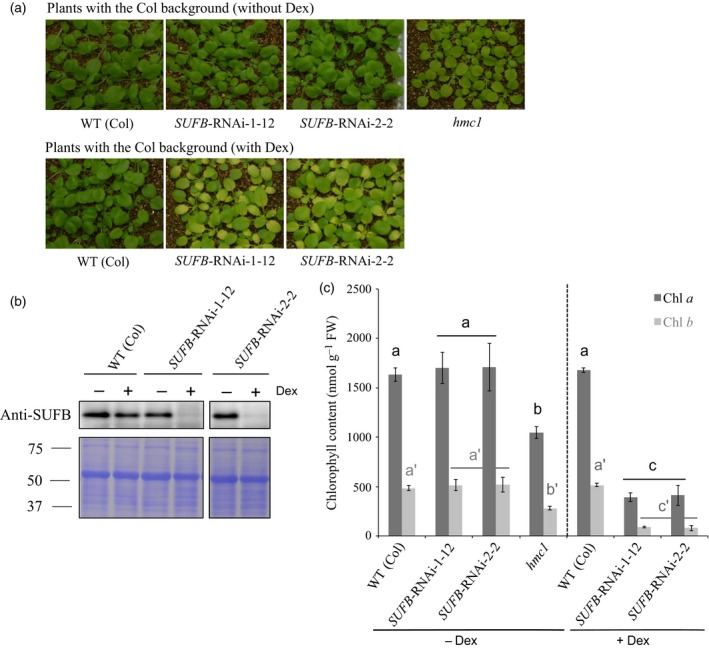
Phenotype of 4‐week‐old SUFB‐deficient plants grown on soil under long‐day conditions. Conditional *SUFB*‐silenced lines (*SUFB‐* RNAi‐1‐12 and 2‐2) were analysed with and without 10 μm dexamethasone (Dex) together with the SUFB‐deficient *hmc1* mutant. (a) Phenotype of SUFB‐deficient plants. (b) SUFB protein levels in SUFB‐deficient mutant and transgenic lines. Total protein extracts (8 μg protein) of developing leaves were analyzed by immunoblotting using anti‐SUFB antiserum (upper row) and the membrane was subsequently stained by Coomassie Brilliant Blue (CBB) as a loading control (bottom row). (c) Chlorophyll *a* and *b* content of developing leaves of SUFB‐deficient plants. Data points represent the mean ± SD of four biological replicates. Letters in black (chlorophyll *a*) or in grey (chlorophyll *b*) above each bar indicate significant differences (*P *<* *0.05) by Tukey's multiple‐comparison test.

The two SUFB‐deficient mutants characterized to date, *hmc1* and *laf6*, show rather different phenotypes with respect to chlorophyll biosynthesis and phytochrome responses. In the absence of available knock‐out lines due to embryo lethality, we constructed conditional *SUFB‐*silencing lines to further investigate the impact of *SUFB* depletion on these responses. In these lines, a dexamethasone (Dex)‐inducible promoter drives the expression of artificial sequences that are complementary to part of the Arabidopsis *SUFB* gene, leading to degradation of *SUFB* mRNA (see Experimental Procedures). It is possible that an RNAi construct suppresses some off‐target gene expression, and we therefore tested two independent RNAi constructs (*SUFB*‐RNAi‐1 and *SUFB*‐RNAi‐2) that were designed to interact with two different sequences within the *SUFB* gene. In agreement with previous SUFB‐deficient lines, both *SUFB* RNAi lines showed a pale green phenotype in 4‐week‐old plants that was dependent on Dex treatment (Figure 1a). The pale phenotype of the *SUFB* RNAi lines correlated with the Dex‐dependent loss of SUFB protein in these lines (Figure 1b). Analysis of chlorophyll levels showed that both RNAi lines were equally depleted in chlorophyll *a* and *b* and that the observed phenotype was stronger than that seen for the *hmc1* mutant (Figure 1c), which shows a moderate reduction in chlorophyll levels in agreement with a previous report (Nagane *et al*., 2010). Identical results were obtained from 7‐day‐old mutant and *SUFB* RNAi seedlings grown on agar plates (Figure S2). Together, these results confirm that a reduction in SUFB leads to reduced chlorophyll accumulation.

### The SUFBC_2_D complex is required for chlorophyll synthesis

**Figure 2:**
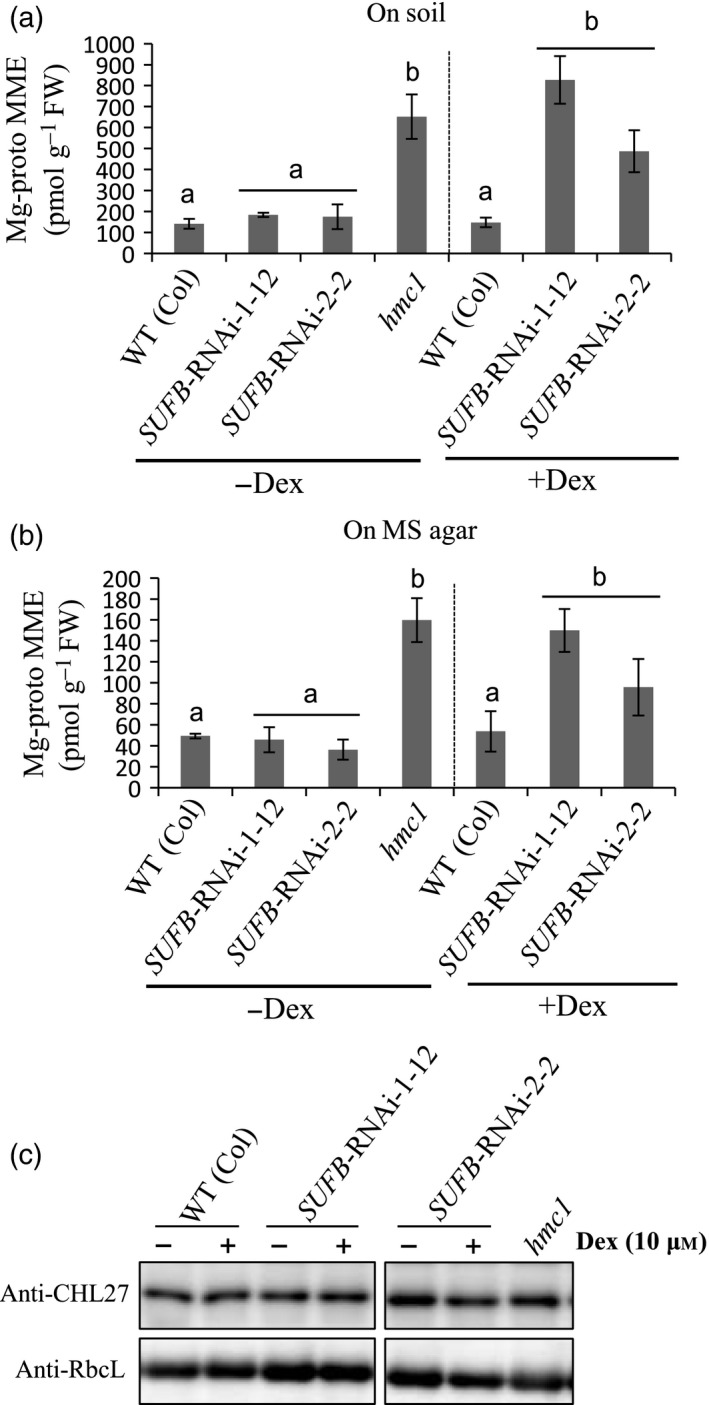
Analysis of chlorophyll biosynthetic intermediates in SUFB‐deficient plants. Conditional *SUFB*‐silenced lines (*SUFB‐* RNAi‐1‐12 and 2‐2) were analysed with and without 10 μm dexamethasone (Dex) together with the SUFB‐deficient *hmc1* mutant. Mg‐protoporphyrin IX monomethylester (Mg‐proto MME) content in (a) developing leaves of 4‐week‐old plants grown on soil under long‐day conditions or (b) in 7‐day‐old seedlings grown on 1/2 MS medium under long‐day conditions. Data points represent the mean ± SD of four biological replicates. Letters above each bar indicate significant differences (*P *<* *0.05) by Tukey's multiple‐comparison test. (c) The abundance of Mg‐proto MME cyclase (CHL27) in SUFB‐deficient mutant and transgenic lines. Total protein extracts (8 μg protein) of developing leaves of 4‐week‐old plants grown on soil under long‐day conditions were analysed by immunoblotting using anti‐CHL27 antisera. Anti‐Rubisco large subunit (RbcL) antisera were used to detect RbcL as the loading control.

To determine how depletion of SUFB affects chlorophyll biosynthesis we measured the levels of key chlorophyll biosynthesis intermediates in the *hmc1* mutant and *SUFB* RNAi lines using high‐performance liquid chromatography (Mochizuki *et al*., 2008). As shown in Figure 2, Mg‐proto MME accumulated in the *hmc1* mutant and the *SUFB* RNAi lines treated with Dex to levels in excess of wild‐type (WT) lines. This was true both for mature plants (Figure 2a) and seedlings (Figure 2b). In contrast, all SUFB‐deficient lines had reduced levels of Proto IX and showed reductions in Mg‐proto (Figure S3). These results indicate that Mg‐proto MME cyclase activity is likely to be compromised in the *hmc1* mutant and the conditional *SUFB* RNAi lines because Arabidopsis mutants that are defective in Mg‐proto MME cyclase activity accumulate this substrate (Tottey *et al*., 2003; Peter *et al*., 2009). As levels of Mg‐proto MME cyclase protein are unaltered in SUFB‐deficient plants (Figure 2c) we conclude that this effect is at the level of Mg‐proto MME cyclase activity. In addition, we interpret the lower levels of Proto IX and Mg‐proto to be due to feedback inhibition in the tetrapyrrole pathway. A blockage in the cyclase step could result in more porphyrin being directed towards heme, leading to inhibition of ALA synthesis (Cornah *et al*., 2003). It is also possible that oxidative stress effects are impairing tetrapyrrole biosynthesis via an alternative mechanism (Schlicke *et al*., 2014).

**Figure 3:**
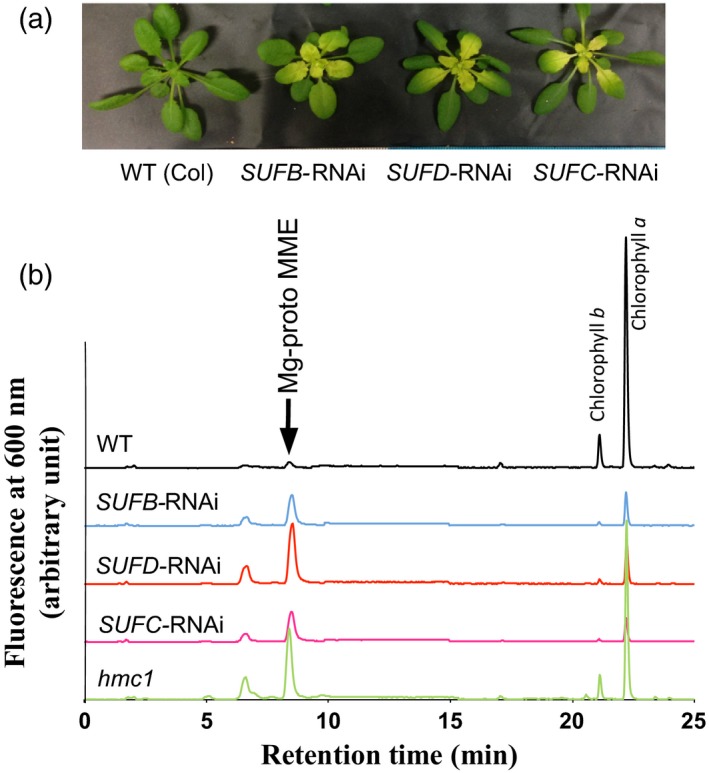
The phenotype of *SUFB*‐, *SUFC*‐ and *SUFD*‐deficient plants. Conditional *SUFB*‐, *SUFC*‐ and *SUFD*‐silenced lines were analyzed after treatment with 10 μm dexamethasone (Dex) together with the SUFB‐deficient *hmc1* mutant. (a) Phenotype of 4‐week‐old wild‐type (WT) and transgenic plants 7 days after Dex treatment. (b) The HPLC profiles of pigments extracted by acetone from young leaves of WT and transgenic plants after Dex treatment. The *x*‐axis indicates retention time. The *y*‐axis indicates fluorescence intensity at 600 nm which was excited at 415 nm in arbitrary units. Mg‐proto MME, Mg‐protoporphyrin IX monomethyl ester.

The effect of SUFB deficiency could be due to an inhibition of Fe–S cluster synthesis or to an independent function of the SUFB protein. To test this we analyzed the phenotype of conditional RNAi‐silencing lines for *SUFD* and *SUFC* (Hu *et al*., 2017). Both *SUFC* and *SUFD* RNAi lines showed pale‐green leaves and were similar in appearance to *SUFB* lines (Figure 3a). In addition, in each case Mg‐proto MME accumulated to a similar extent to that observed for the *SUFB* RNAi line or *hmc1* (Figure 3b). These results therefore indicate that accumulation of Mg‐proto MME is due to an impaired function of the SUFBC_2_D complex and not to a unique function of SUFB.

### Overexpression of SUFB complements the white‐light‐grown phenotype of the *laf6* mutant

**Figure 4:**
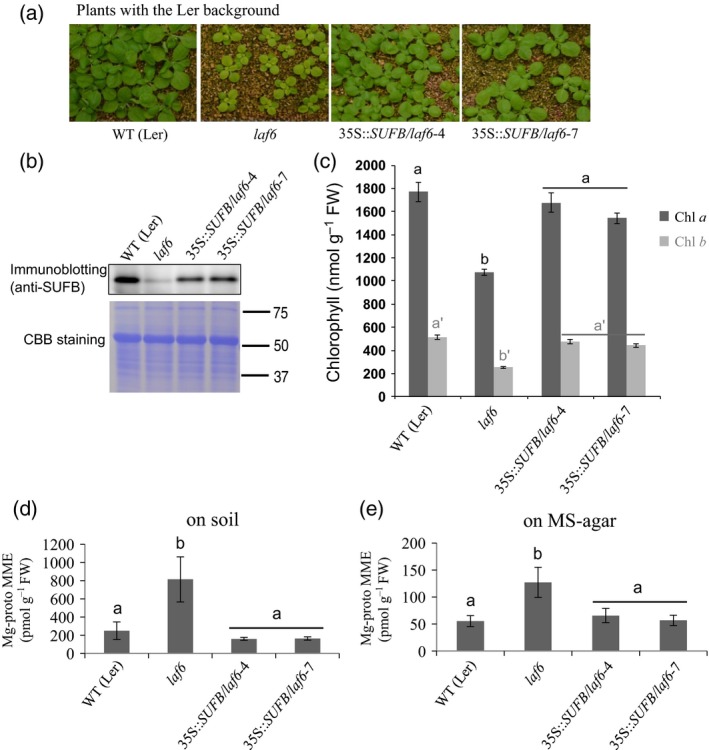
Complementation of *laf6* with *SUFB*. (a) Comparison of *laf6* and *SUFB* overexpressing lines in a *laf6* background grown on soil for 3 weeks under long‐day conditions. (b) SUFB protein levels in the mutant and transgenic lines. Total protein extracts (8 μg protein) of developing leaves were analyzed by immunoblotting using anti‐SUFB antiserum (upper row) and the membrane was subsequently stained by Coomassie Brilliant Blue (CBB) as a loading control (bottom row). (c) Chlorophyll *a* and *b* content of developing leaves of mutant and transgenic plants. (d) Mg‐protoporphyrin IX monomethylester (Mg‐proto MME) content in developing leaves of 4‐week‐old plants grown on soil under long‐day conditions. (e) The Mg‐proto MME content in 7‐day‐old seedlings grown on 1/2 MS medium under long‐day conditions. Data points represent the mean ± SD of four biological replicates. Letters in black or grey above each bar indicate significant differences (*P *<* *0.05) by Tukey's multiple‐comparison test.

To determine whether all the phenotypes observed in the *laf6* mutant allele were due to SUFB deficiency, we generated several independent transgenic lines that expressed 35S::*SUFB* in the *laf6* mutant, which is in the Landsberg *erecta* (L*er*) background. Two overexpression lines (35S::*SUFB*/*laf6*‐4 and ‐7) were selected and used for further analysis (Figure 4a). The *laf6* mutant shows a reduced level of the SUFB protein (to 15% of the WT (L*er*) level), and this was increased in the two *SUFB*‐complemented lines, although the abundance of SUFB was still less than WT, at 64 and 70% of the WT level, respectively (Figure 4b). Nevertheless, both 35S::*SUFB* lines restored the visibly pale phenotype of 4‐week‐old *laf6* plants to a WT appearance (Figure 4a), and this was confirmed by an analysis of chlorophyll levels. As shown in Figure 4(c), chlorophyll was significantly reduced in the *laf6* mutant in agreement with Møller *et al*. (2001), but levels of both chlorophyll *a* and chlorophyll *b* were similar to WT levels in the 35S::*SUFB*/*laf6*‐4 and ‐7 lines. Identical results were observed in 7‐day‐old seedlings (Figure S4).

Next we examined the chlorophyll biosynthesis intermediates Proto IX, Mg‐proto and Mg‐proto MME. In agreement with what we observed in the *hmc1* mutant and *SUFB* RNAi lines (Figure 2), the *laf6* mutant also showed elevated levels of Mg‐proto MME in 4‐week‐old plants (Figure 4d) and in seedlings (Figure 4e). This aspect of the *laf6* phenotype was fully complemented by overexpression of SUFB (Figure 4d,e). Similarly to other SUFB‐deficient lines, *laf6* also appeared to have slightly lower levels of Proto IX and Mg‐proto; however, this was not significant in this case (Figure S5).

### Proto IX accumulation and long hypocotyl in far‐red‐light phenotypes of *laf6* are not complemented by overexpression of SUFB

**Figure 5:**
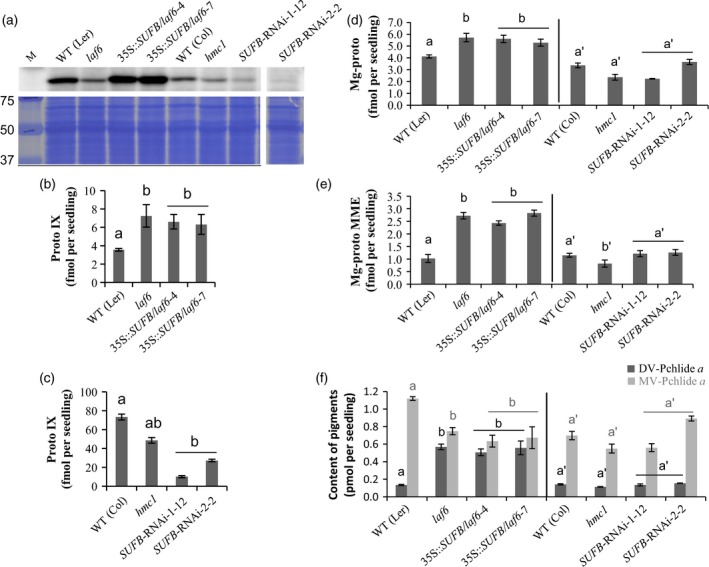
The phenotype of dark‐grown SUFB‐deficient seedlings. Comparison of 7‐day‐old etiolated seedlings of conditional *SUFB*‐silenced lines, *hmc1*,* laf6* and *laf6* complemented lines expressing SUFB grown on 1/2 MS medium with 0.8% agar and 1% sucrose. (a) Levels of SUFB protein in mutant and transgenic seedlings. Total protein extracts (16 μg protein) were analyzed by immunoblotting using anti‐SUFB antiserum (upper row) and the membrane was subsequently stained by Coomassie Brilliant Blue (CBB) as a loading control (lower row). (b, c) Protoporphyrin IX (Proto IX) content in etiolated seedlings in the L*er* (b) and Col (c) backgrounds. Data points represent the mean ± SD of three biological replicates. Letters above each bar indicate significant differences (*P *<* *0.05) by Tukey's multiple‐comparison test. (d) Mg‐proto IX (Mg‐proto) and (e) Mg‐proto IX monomethyl ester (Mg‐proto MME) contents of mutant and transgenic seedlings. Data points represent the mean ± SD of three biological replicates. Letters in black (Mg‐proto) or in grey (Mg‐proto MME) above each bar indicate significant differences (*P *<* *0.05) by Tukey's multiple‐comparison test. (f) 3,8‐Divinyl protochlorophyllide *a* (DV‐Pchlide *a*) or monovinyl protochlorophyllide *a* (MV‐Pchlide *a*) contents in mutant and transgenic seedlings. Data points represent the mean ± SD of three biological replicates. Letters in black (DV‐Pchlide *a*) or in grey (MV‐Pchlide *a*) above each bar indicate significant differences (*P *<* *0.05) by Tukey's multiple‐comparison test.

The *laf6* phenotype was originally characterized by an accumulation of Proto IX in dark‐grown and FR light‐grown seedlings (Møller *et al*., 2001). We therefore measured the levels of Proto IX and other chlorophyll precursors in 7‐day‐old WT and SUFB‐deficient etiolated seedlings (Figure 5). First, we confirmed SUFB protein levels in dark‐grown seedlings of all the lines used. As shown in Figure 5(a), the *laf6* mutant contained reduced amounts of SUFB compared with WT (L*er*) and the two complemented lines over‐accumulated SUFB. Only a small decrease in SUFB was observed in *hmc1*, while in the *SUFB* RNAi lines, SUFB protein levels were severely reduced by Dex treatment. These results are consistent with those seen in 4‐week‐old plants (Figures 1b and 4b).

As reported by Møller *et al*. (2001), dark‐grown *laf6* seedlings showed elevated levels of Proto IX compared with WT (L*er*) seedlings (Figure 5b). In addition, Mg‐proto, Mg‐proto MME (Figure 5d,e) and DV‐Pchlide (Figures 5f and S6) all showed increased levels in *laf6* compared with WT (L*er*). In contrast, neither the *hmc1* mutant nor the *SUFB* RNAi lines showed elevated chlorophyll precursors in dark‐grown seedlings (Figures 5c–f and S6). In fact, *hmc1* and the *SUFB*‐silenced etiolated seedlings accumulated less Proto IX than did WT (Col) seedlings (Figure 5c). In addition, overexpression of SUFB did not rescue the increase in chlorophyll precursors seen in *laf6* (Figures 5b,d–f and S6). Taken together, these results show that the excessive accumulation of Proto IX, Mg‐proto, Mg‐proto MME and DV‐Pchlide in etiolated *laf6* seedlings is not the consequence of altered SUFB levels in this mutant.

**Figure 6:**
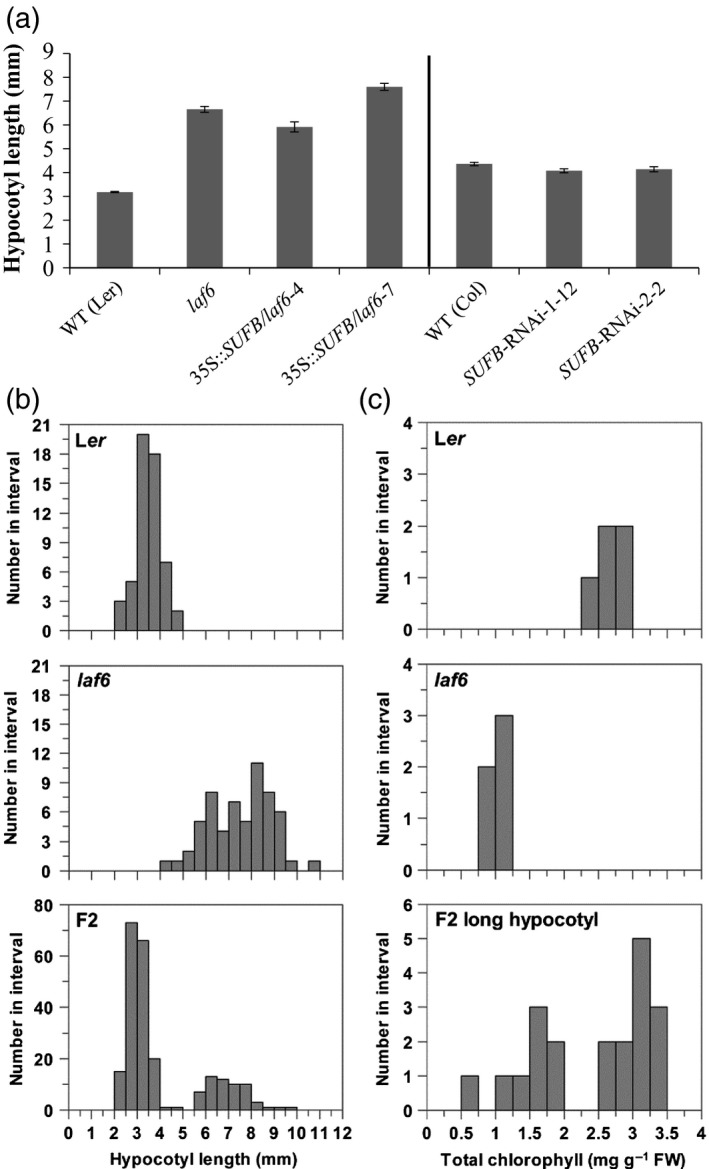
Segregation of the *laf6* long‐hypocotyl phenotype. (a) Hypocotyl length of *laf6*,* laf6* expressing *SUFB* and conditional SUFB‐deficient seedling grown for 6 days under far‐red (FR) light. Data shown are mean ± SD of four biological replicates. (b, c) Segregation of the long‐hypocotyl and pale leaf phenotypes of back‐crossed *laf6* seedlings. (b) Histogram of hypocotyl lengths of L*er*,* laf6* and the F_2_ generation (L*er* × *laf6*) seedlings after 6 days’ growth in FR light. (c) Histogram of the foliar chlorophyll content of the parent lines and 20 randomly selected F_2_ seedlings with long hypocotyls after 6 days under FR. Following the FR treatment, all seedlings were supplemented with 3% sucrose and recovered in low‐intensity white light (WL) (4 days at 5 μmol m^−2^ sec^−1^ followed by 2 days at 25 μmol m^−2^ sec^−1^) before being transferred to WL (100 μmol m^−2^ sec^−1^) for 11 days.

The *laf6* mutant was originally isolated from a screen for mutants with long hypocotyl after growth under FR light (Møller *et al*., 2001). To investigate this response and further explore the link between Fe–S cluster biosynthesis and phytochrome signaling, the response of the *SUFB* mutant and transgenic lines to FR light was examined. As expected, *laf6* had a longer hypocotyl than the WT when grown under FR light irradiation (Figure 6a). However, this response was not rescued in the *SUFB*‐complemented lines, and the *SUFB* RNAi lines did not show enhanced hypocotyl elongation compared with WT (Col) seedlings on Dex (Figure 6a). These results show that the reduced responsiveness toward FR light in *laf6* is not correlated with SUFB levels in seedlings.

### The long hypocotyl phenotype of *laf6* is due to a second, segregating mutation

The results in our hypocotyl elongation assays and on the accumulation of Proto IX and DV‐Pchlide suggest that the *laf6* mutant has an additional mutation causing that phenotype independently of its effect on SUFB protein levels. To examine this possibility, we analyzed the segregation of *laf6* phenotypes after backcrossing *laf6* with WT (L*er*). Seedlings in the F_2_ generation were analyzed initially for hypocotyl length in FR light and subsequently for chlorophyll biosynthesis in white light (Figure 6b,c). F_2_ seedlings segregated into two populations with longer (>5.5 mm) and shorter (<5.0 mm) hypocotyls. Of 234 seedlings measured, 176 (75.2%) had short hypocotyls while 58 (24.8%) had long hypocotyls, a ratio indicating that hypocotyl length was controlled by a single recessive locus (Figure 6b). Twenty seedlings from the population with long hypocotyls were then grown under continuous white light and chlorophyll levels determined. Again two populations were observed with 12 (60%) showing higher chlorophyll levels [>2.5 mg g^−1^ fresh weight (FW)] similar to the level seen in WT seedlings and 8 (40%) with lower chlorophyll levels (<2.0 mg g^−1^ FW) consistent with *laf6* seedlings (Figure 6c). These results indicate that the long‐hypocotyl phenotype under FR light and the low‐chlorophyll phenotype under white light are controlled by two independent genes. This is consistent with our data showing that the long‐hypocotyl phenotype is not due to SUFB deficiency.

The *laf6* mutant shows an accumulation of Proto IX and other chlorophyll precursors in dark‐grown seedlings, and we speculated that the long‐hypocotyl phenotype might be linked to a deficiency in synthesis of the phytochrome chromophore phytochromobilin, which is synthesized from heme (Frankenberg‐Dinkel and Terry, 2009; see Figure S1). This was initially examined using the chromophore analogue phycocyanobilin (PCB; Elich *et al*., 1989) and it was concluded that *laf6* was not compromised in phytochrome chromophore synthesis (Møller *et al*., 2001). However, it was subsequently shown that the altered chromophore structure of PCB cannot support a FR high‐irradiance response leading to inhibition of hypocotyl elongation (Hanzawa *et al*., 2002), so we re‐addressed this question by feeding the chromophore precursor biliverdin IXα. However, as shown in Figure S7, biliverdin IXα did not rescue the long‐hypocotyl phenotype of *laf6*, but could rescue a *hy1* mutant control.

## Discussion

### Iron–sulfur clusters are required for chlorophyll synthesis

Mutants lacking SUFB are reduced in PSI subunit accumulation and have reduced chlorophyll levels (Hu *et al*., 2017). However, it was unclear whether the reduction in chlorophyll was a consequence of a reduced accumulation of photosynthetic proteins or whether chlorophyll synthesis was directly affected. Here we show that a reduction of SUFB protein levels results in excessive accumulation of the chlorophyll precursor Mg‐proto MME and an overall reduction in chlorophyll levels. Both the *laf6* and *hmc1*, the SUFB‐deficient mutant alleles showed this phenotype as did the *SUFB* RNAi knock‐down lines. Moreover, the *SUFC* and *SUFD* RNAi lines both accumulated Mg‐proto MME, suggesting that all subunits of the SUFBC_2_D complex are necessary for optimal chlorophyll synthesis and that inhibition of Fe–S cluster biosynthesis is the critical factor. To date no chlorophyll *a* biosynthesis enzymes have been shown to be Fe–S proteins, although two enzymes involved in chlorophyll metabolism, 7‐hydroxymethyl‐chlorophyll *a* reductase and pheophorbide *a* oxygenase, contain Fe–S clusters (Pruzinská *et al*., 2003; Meguro *et al*., 2011; Wang and Liu, 2016) and the substrates of both of these enzymes also accumulate in the *hmc1* mutant (Nagane *et al*., 2010) (see Figure S1). It is also reported that siroheme biosynthesis requires an Fe–S protein, sirohydrochlorin ferrochelatase (Saha *et al*., 2012), although the effect of Fe–S deficiency on siroheme biosynthesis is not clear at present. Fe–S proteins, such as ferredoxin, could also be involved, but this has only been established for chlorophyll *a* oxygenase (Oster *et al*., 2000) and heme oxygenase (Muramoto *et al*., 2002). Mutants in both of these enzymes are pale in color (Chory *et al*., 1989; Terry, 1997; Espineda *et al*., 1999) and thus could be candidate targets for an impact of Fe–S cluster deficiency.

In the current study we have demonstrated that Mg‐proto MME accumulates in SUFB‐deficient mutants. Accumulation of Mg‐proto MME is seen in mutants that lack Mg‐proto MME cyclase activity (Tottey *et al*., 2003; Peter *et al*., 2009; Hollingshead *et al*., 2012), and we therefore propose that Mg‐proto MME cyclase activity is impaired in SUFB‐deficient plants, as the protein level of cyclase is unaffected. Based on genetic and biochemical analysis in barley (Rzeznicka *et al*., 2005), Mg‐proto MME cyclase is predicted to contain three subunits, although only two, YCF54 and CLH27, have been identified (Tottey *et al*., 2003; Albus *et al*., 2012; Hollingshead *et al*., 2012; Bollivar *et al*., 2014). However, neither of these subunits have Fe–S cluster‐binding motifs (Tottey *et al*., 2003; Hollingshead *et al*., 2012). It is therefore possible that the third as yet unidentified cyclase subunit contains an Fe–S cluster. Alternatively, other Fe–S proteins may function to accept electrons from the cyclase, as the cyclase reaction requires an electron acceptor. We have previously observed that treatment of methylviologen, an inhibitor of PSI, causes accumulation of Mg‐proto MME in cucumber seedlings (Aarti *et al*., 2006). Therefore, it is possible that PSI, which contains three Fe–S clusters, is itself required for the cyclase reaction. Finally, it is possible that the regulation of Mg‐proto MME cyclase is indirectly impaired in SUFB‐deficient plants. Little is known about the regulation of Mg‐proto MME cyclase, except for the involvement of NADPH‐dependent thioredoxin reductase C (NTRC). A mutant lacking this enzyme accumulates Mg‐proto MME (Stenbaek *et al*., 2008), thus it is possible that the SUFB‐deficient plants analyzed in this study are also impaired in the redox network in which NTRC is involved.

### The phenotype of *laf6* is partially due to second site mutations

When chlorophyll precursors were measured in dark‐grown seedlings we observed differences between the *laf6* allele and other SUFB‐deficient lines. The *hmc1* mutant and *SUFB* RNAi lines showed moderate decreases in Proto IX, Mg‐proto and Mg‐proto MME, although this was not significant in all cases, and they did not have reduced Pchlide levels. In contrast, the *laf6* mutant accumulated Proto IX, Mg‐proto, Mg‐proto MME and DV‐Pchlide and showed a decrease in MV‐Pchlide in dark‐grown seedlings. The accumulation of Proto IX was consistent with a previous report (Møller *et al*., 2001). It is difficult to explain this set of results, but one possibility is that the conversion of DV‐Pchlide into MV‐Pchlide is impaired in the *laf6* background and the general increase in porphyrins could be due to a regulatory effect such as product inhibition for enzymes prior to DV‐Pchlide in the pathway. Importantly, with regard to the function of SUFB, the effect of the *laf6* mutation on porphyrin accumulation in dark‐grown seedlings could not be rescued by overexpression of SUFB, in contrast to the light‐dependent phenotypes of *laf6*. These results therefore indicate that the increase in chlorophyll intermediates in dark‐grown *laf6* seedlings is caused by a second site mutation in the *laf6* background.

In the original description of *laf6*, the accumulation of Proto IX was linked to a long‐hypocotyl phenotype under FR light (Møller *et al*., 2001). The authors had speculated that perturbations to tetrapyrrole synthesis might lead to an inhibition of phytochrome chromophore synthesis. We also tested this hypothesis, partly because more recent evidence indicated that the original control used could not have been successful (Hanzawa *et al*., 2002), but saw no evidence that the long‐hypocotyl phenotype was caused by chromophore deficiency. None of the other SUFB‐deficient lines tested had a long hypocotyl in FR light, and the phenotype was not complemented by SUFB expression. Moreover, segregation analysis following a backcross to WT seedlings demonstrated that the long‐hypocotyl phenotype of *laf6* was separable from the chlorophyll‐deficient phenotype of SUFB‐deficient plants, and therefore also due to a second site mutation. It is not known whether the accumulation of porphyrins in dark‐grown seedlings and the long‐hypocotyl phenotype in FR light are linked. Both processes are regulated by phytochrome (Whitelam *et al*., 1993; McCormac and Terry, 2002), but while hypocotyl elongation is inhibited by phytochrome, porphyrin synthesis is promoted.

In conclusion, we have shown that deficiency in Fe–S cluster biosynthesis leads to reduced chlorophyll accumulation and we have identified Mg‐proto MME cyclase activity as the most likely target of inhibition. This is the first step in understanding the role of Fe–S cluster biosynthesis in chlorophyll metabolism and provides important clues about the mechanism of Mg‐proto MME cyclase. In addition, we have resolved the discrepancy between the phenotypes of different *SUFB* mutant alleles and demonstrated that the long‐hypocotyl phenotype of *laf6* is unrelated to deficiency in SUFB. Together, these results help clarify the role of Fe–S clusters in plant development.

## Experimental Procedures

### Plant materials

The *Arabidopsis thaliana laf6* and *hmc1* mutants were described by Møller *et al*. (2001) and Nagane *et al*. (2010), respectively. Sterilized Arabidopsis seeds were sown on half‐strength Murashige and Skoog (1/2 MS) medium containing 1% (w/v) sucrose and 0.8% (w/v) agar or soil. Seeds were kept in the dark at 4°C for 3 days to induce uniform germination. The plants were subsequently grown under long‐day (16‐h light/8‐h dark) growth conditions under fluorescent light (70–90 μmol m^−2^ sec^−1^ at 23°C). For pigment and immunoblotting analyses, either developing leaves of 4‐week‐old plants or the whole seedlings of 7‐day‐old plants were harvested. For experiments under FR light (6 μmol m^−2^ sec^−1^) and in the dark, seedlings were grown for 7 days on 1/2 MS agar plates at 23°C.

For segregation experiments, surface sterilized seeds (L*er*,* laf6*, F_2‐1_ and F_2‐2_) were sown on 1/2MS in 1% (w/v) agar, pH 5.8. After a 3‐day cold treatment (in the dark at 4°C), seeds were transferred to white light (WL; 100 μmol m^−2^ sec^−1^, 23°C) for 2 h, then incubated in the dark (23°C) for 1 day. Seedlings were then grown for 6 days in FR light (23°C). After the FR treatment, seedlings were gently flattened onto the surface of the medium and photographed for analysis of hypocotyl length. During this process, plates were kept in very low‐intensity WL as much as possible. Twenty randomly selected seedlings displaying a long‐hypocotyl phenotype in FR light were then transferred to a separate plate. All seedlings were then supplemented with filter‐sterilized 3% (w/v) sucrose and recovered in low‐intensity WL (4 days at 5 μmol m^−2^ sec^−1^ followed by 2 days at 25 μmol m^−2^ sec^−1^) before being transferred to WL (100 μmol m^−2^ sec^−1^) for 11 days. Chlorophyll assays were performed on five seedlings of each parent line, as well as the 20 randomly selected seedlings with long hypocotyls after the FR treatment.

### Cloning and Arabidopsis transformation

For *laf6* complementation, a complementary DNA (cDNA) covering the coding region of Arabidopsis *SUFB* was cloned from Arabidopsis (Col ecotype) into pEarleyGate100 (Earley *et al*., 2006) as described in the companion paper (Hu *et al*., 2017). In brief, to generate Dex‐inducible RNAi constructs, two 300‐bp regions from the coding sequence of either *SUFB, SUFD* or *SUFC* were amplified by PCR using the primer sets described in Hu *et al*. (2017). The PCR products were cloned into the pENTR4 dual selection vector (ThermoFisher Scientific, http://www.thermofisher.com), the resulting entry clones were recombined with the pOpOff2 (kan) vector (Wielopolska *et al*., 2005) by LR Clonase II (ThermoFisher Scientific) recombination reactions. the whole construct was introduced into *Agrobacterium tumefaciens* strain GV3101 and used to infect Arabidopsis with the floral dip method (Clough and Bent, 1998). Two‐week‐old seedlings of transformants with the pEarleyGate100 vector were selected by spraying with BASTA and transformants containing the pOpOff2 (kan) vector were selected on 50‐μm kanamycin on 1/2 MS plates. Third‐generation homozygous plants were employed for further analysis.

### *SUFB*,* SUFC* and *SUFD* RNAi induction

Dex was first dissolved in ethanol to a concentration of 20 mm, and then diluted using 0.02% Tween‐20 aqueous solution to give a final concentration of 10 μm. To induce RNAi‐mediated gene silencing, 3‐week‐old plants grown in soil were sprayed with 10 μm Dex solution. In control experiments, plants were sprayed with the aqueous solution without Dex, but containing the same concentration of ethanol and Tween‐20. To induce *SUFB*‐silencing in plants grown on 1/2 MS medium, ethanol, with or without Dex, was added to autoclaved 1/2 MS medium (at about 55°C) to a final concentration of 10 μm. It is noted that the effects of RNAi suppression were only visible in developing leaves in mature 3‐ or 4‐week‐old plants. Therefore, only developing leaves were harvested for protein and pigment analyses.

### Pigment analysis

For the segregation analysis, leaf tissue was weighed and then extracted in ice‐cold 80% acetone. Samples were centrifuged at 16 100 ***g*** for 5 min, and the absorbance of the supernatant was determined at 647 nm and 663 nm using a U‐2001 spectrophotometer (Hitachi, http://www.hitachi.com/). Total chlorophyll content (μg mL^−1^) was calculated by combining the chlorophyll *a* (12.25A_663_–2.79A_647_) and chlorophyll *b* (21.5A_647_–5.1A_663_) values before conversion to mg g^−1^ FW. For other experiments, chlorophyll and its intermediates were extracted from leaf tissue by homogenization with acetone, which was pre‐cooled to −30°C to suppress endogenous chlorophyllase activity (Hu *et al*., 2013). Extracts were subsequently centrifuged for 5 min at 20 000 ***g*** at 4°C, and the supernatant was analyzed by HPLC using a symmetry C8 column (150 mm long, 4.6 mm ID; Waters, http://www.waters.com/) according to the method of Zapata *et al*. (2000). Chlorophyll concentrations were estimated from the absorption monitored at 410 nm. Chlorophyll *a* and *b* standards were purchased from Juntec Co. Ltd ( http://juntec.co.jp/) and pheophorbide *a* was purchased from Wako Pure Chemical Industries, Ltd ( http://www.wako-chem.co.jp/english/). A fluorescence detector (Hitachi) was used for the detection of Proto IX, Mg‐proto, and Mg‐proto MME *a* after separation by HPLC. Proto IX was detected at 634 nm following excitation at 400 nm, Mg‐proto and Mg‐proto MME were detected at 600 nm after excitation at 417 nm, while DV‐Pchlide *a* and MV‐Pchlide *a* were quantified by measuring absorbance at 439 nm.

### Immunoblot analysis

Total protein was extracted from leaves using 10 volumes (v/w) of protein extraction buffer containing 50 mm 2‐amino‐2‐(hydroxymethyl)‐1,3‐propanediol (TRIS)‐HCl (pH 8.0), 12% (w/v) sucrose (Suc), 2% (w/v) lithium lauryl sulfate and 1.5% (w/v) dithiothreitol. The protein concentration of samples was determined using the Rc‐Dc protein assay kit (Bio‐Rad, http://www.bio-rad.com/). Before SDS‐PAGE separation, all samples were mixed with an equal volume of 2× urea buffer containing 10 mm TRIS‐HCl (pH 8.0), 10% (w/v) Suc, 2% (w/v) SDS, 1 mm EDTA, 4 mm dithiothreitol, 0.04% (w/v) bromophenol blue and 10 m urea. Samples were then separated on a 14% polyacrylamide gel and electro‐blotted to polyvinylidene difluoride (PVDF) membranes. SUFB protein was detected using an anti‐SUFB antiserum raised against recombinant Arabidopsis SUFB protein expressed in *E. coli* (Rossetta DE3, Merck, http://www.merck.com/). The antibody raised against Arabidopsis CHL27 was a kind gift from Professor Sabeeha Merchant (University of California, Los Angeles, CA, USA).

### Statistical analysis

Samples from two ecotypes (L*er* and Col) were analyzed separately. The WT, mutants and the transgenic lines in each ecotype were first analyzed as explanatory valuables by generalized linear mixed‐effects models (GLMMs). Two *SUFB‐*complementation lines (35S::*SUFB/laf6‐*4 and ‐7) and two conditional *SUFB‐*RNAi lines (*SUFB‐*RNAi‐1‐12 and 2‐2) in Figures 2(b), 3, 4, S2 and S4 were nested and treated as random effects variables. After confirming the significance of the GLMMs (*P *<* *0.05), multiple pairwise comparison was made with the Tukey contrasts fit for each result by using the ‘glht()’ function of the ‘multicomp’ package (Hothorn *et al*., 2008) of R (v.3.2.2; R Core Team, 2015), which allows simultaneous tests for outputs of mixed‐effects models.

## Conflict of Interest

The authors declare no conflict of interest.

## Supporting information


**Figure S1.** Tetrapyrrole biosynthetic pathway.

**Figure S2.** Phenotype of 7‐day‐old SUFB‐deficient seedlings grown on 1/2 MS medium under long‐day conditions.

**Figure S3.** Analysis of chlorophyll biosynthetic intermediates in SUFB‐deficient plants.

**Figure S4.** Complementation of *laf6* with *SUFB*.

**Figure S5.** The protoporphyrin IX (a) and Mg‐protoporphyrin IX (b) content of the developing leaves of 4‐week‐old *laf6*‐ and *SUFB*‐overexpressing lines in a *laf6* background grown on soil under long‐day conditions.

**Figure S6.** Detection of protochlorophyllide *a* in 7‐day‐old etiolated mutant and transgenic seedlings with altered SUFB levels.

**Figure S7.** The effects of 0.1 mm biliverdin IXα on hypocotyl length under far‐red light of 6‐day‐old WT, *laf6*,* hy1* and *phyA* seedlings.
