# Supplementary material for: The iron–sulfur cluster biosynthesis protein SUFB is required for chlorophyll synthesis, but not phytochrome signaling
Source: Plant J. 2017 Feb 8;89(6):1184–94. doi: 10.1111/tpj.13455 (PMC5347852; doi:10.1111/tpj.13455)
Supplement: Supplementary file 1 — Figure S1. Tetrapyrrole biosynthetic pathway. [file TPJ-89-1184-s001.pdf]

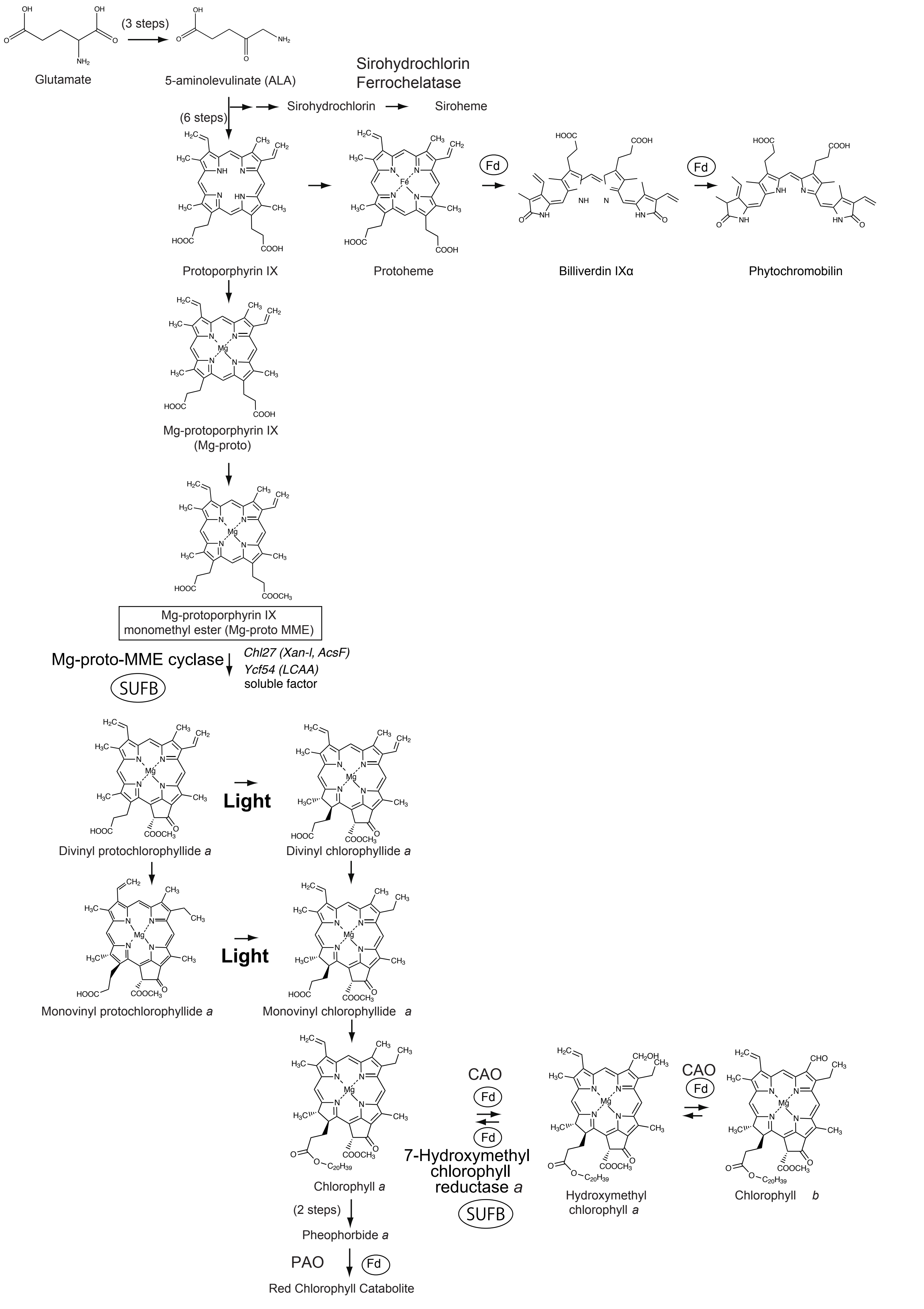

Figure S1. Tetrapyrrole biosynthetic pathway. The enzymatic steps that are proposed to be affected by SUFB deficiency, 7-hydroxymethyl-chlorophyll a reductase (Nagane et al, 2010) and Mg-proto-MME cyclase (this study) are indicated. Steps known to utilize the Fe-S protein ferredoxin (Fd) are also indicated. CAO, chlorophyllide a oxygenase; PAO, pheophorbide a oxygenase.
