# Supplementary material for: The iron–sulfur cluster biosynthesis protein SUFB is required for chlorophyll synthesis, but not phytochrome signaling
Source: Plant J. 2017 Feb 8;89(6):1184–94. doi: 10.1111/tpj.13455 (PMC5347852; doi:10.1111/tpj.13455)
Supplement: Supplementary file 2 — Figure S2. Phenotype of 7‐day‐old SUFB‐deficient seedlings grown on 1/2 MS medium under long‐day conditions. [file TPJ-89-1184-s002.pdf]

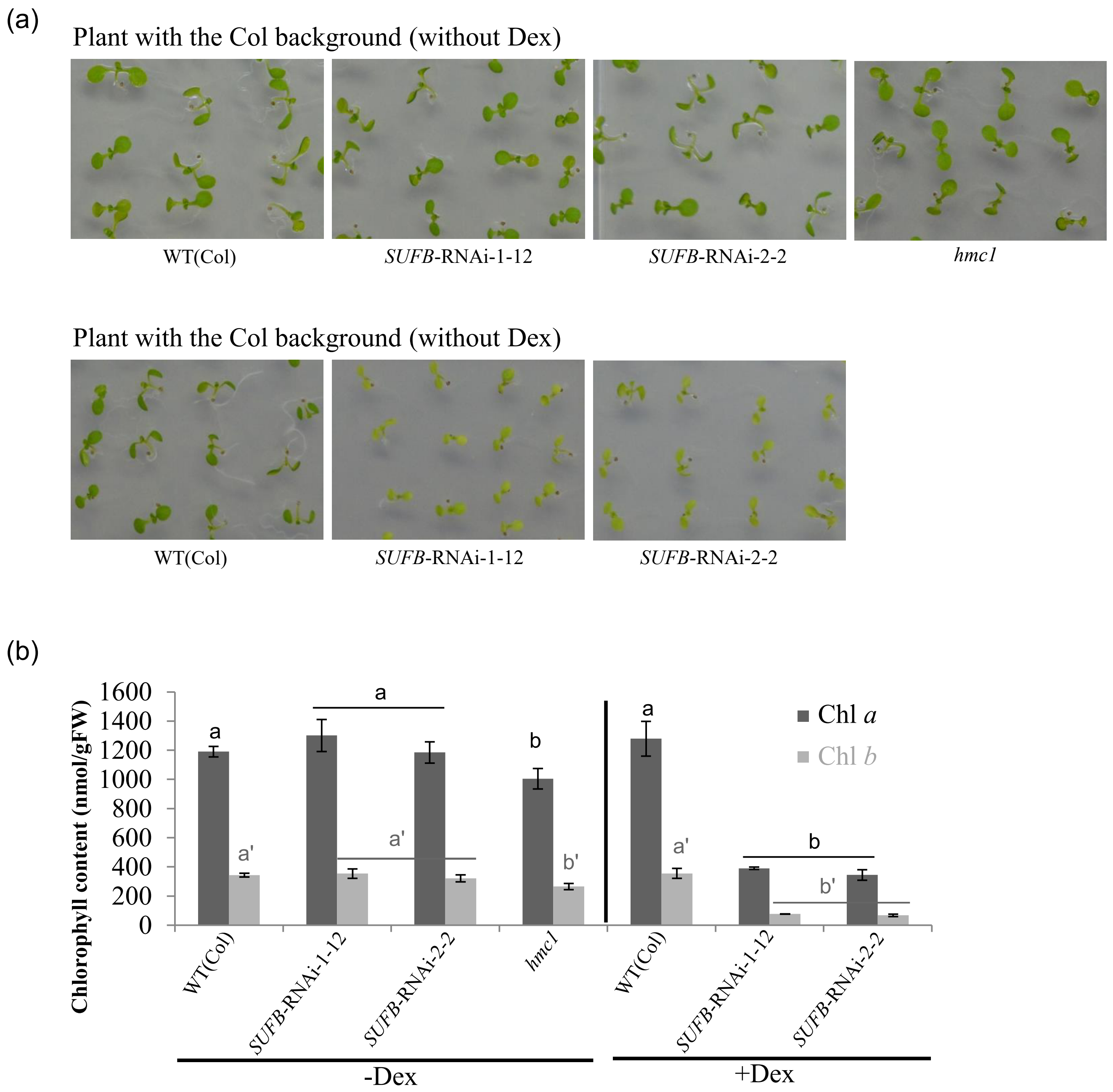

Figure S2. Phenotype of 7-day-old SUFB-deficient seedlings grown on 1/2 MS medium under long-day conditions. (a) Phenotype of SUFB-deficient seedlings. (b) Chlorophyll a and b content of SUFB-deficient seedlings. Data points represent the mean ± SD of four biological replicates. Letters in black (chlorophyll a) or in grey (chlorophyll b) above each bar indicate significant differences ($P < 0.05$) by Tukey's multiple-comparison test.
