# Supplementary material for: The iron–sulfur cluster biosynthesis protein SUFB is required for chlorophyll synthesis, but not phytochrome signaling
Source: Plant J. 2017 Feb 8;89(6):1184–94. doi: 10.1111/tpj.13455 (PMC5347852; doi:10.1111/tpj.13455)
Supplement: Supplementary file 3 — Figure S3. Analysis of chlorophyll biosynthetic intermediates in SUFB‐deficient plants. [file TPJ-89-1184-s003.pdf]

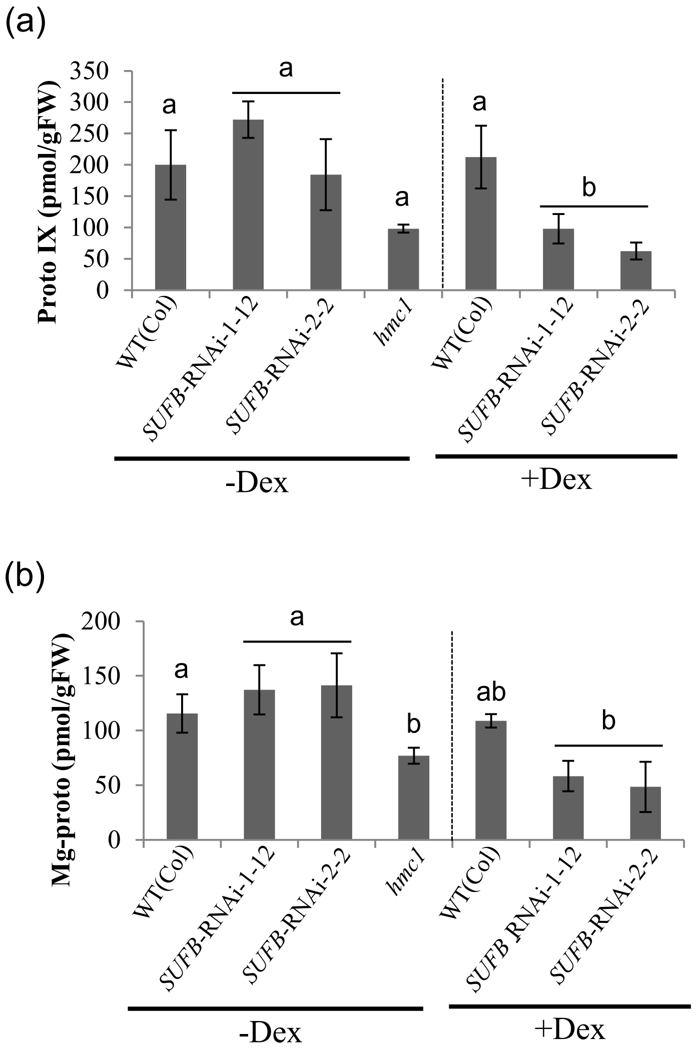

Figure S3. Analysis of chlorophyll biosynthetic intermediates in SUFB-deficient plants. (a) Proto IX and (b) Mg-proto content of the developing leaves of 4-week-old plants grown on soil under long-day conditions. Data points represent the mean ± SD of four biological replicates. Letters above each bar indicate significant differences ($P < 0.05$) by Tukey's multiple-comparison test.
