# Supplementary material for: The iron–sulfur cluster biosynthesis protein SUFB is required for chlorophyll synthesis, but not phytochrome signaling
Source: Plant J. 2017 Feb 8;89(6):1184–94. doi: 10.1111/tpj.13455 (PMC5347852; doi:10.1111/tpj.13455)
Supplement: Supplementary file 4 — Figure S4. Complementation of laf6 with SUFB. [file TPJ-89-1184-s004.pdf]

(a)

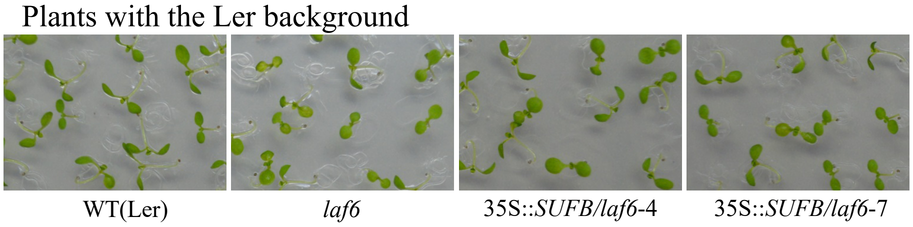


(b)

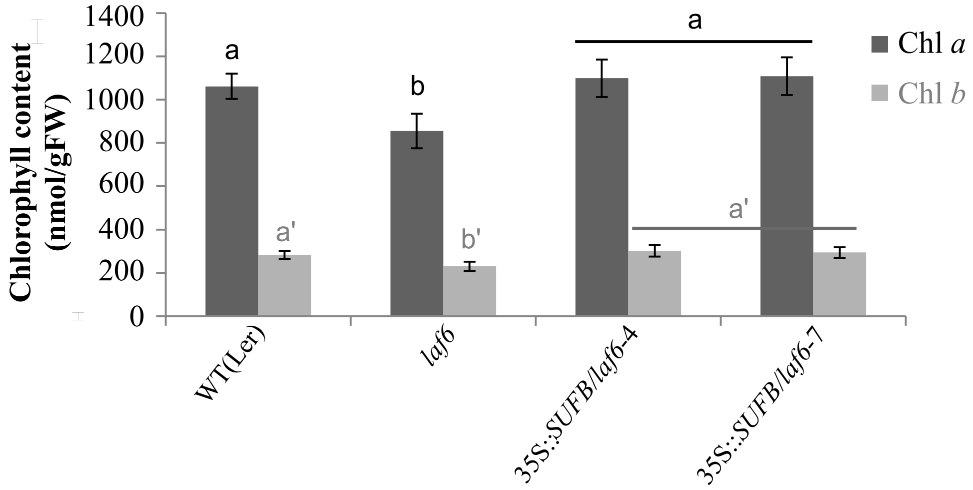


Figure S4. Complementation of laf6 with SUFB. (a) Comparison of 7-day-old seedlings of laf6 and SUFB overexpressing lines in a laf6 background grown on 1/2 MS medium under long day conditions. Chlorophyll a and b content of mutant and transgenic plants. Data points represent the mean ± SD of four biological replicates. Letters in black (chlorophyll a) or in grey (chlorophyll b) above each bar indicate significant differences ($P < 0.05$) by Tukey's multiple-comparison test.
