# Supplementary material for: The iron–sulfur cluster biosynthesis protein SUFB is required for chlorophyll synthesis, but not phytochrome signaling
Source: Plant J. 2017 Feb 8;89(6):1184–94. doi: 10.1111/tpj.13455 (PMC5347852; doi:10.1111/tpj.13455)
Supplement: Supplementary file 5 — Figure S5. The protoporphyrin IX (a) and Mg‐protoporphyrin IX (b) content of the developing leaves of 4‐week‐old laf6‐ and SUFB‐overexpressing lines in a laf6 background grown on soil under long‐day conditions. [file TPJ-89-1184-s005.pdf]

(a)

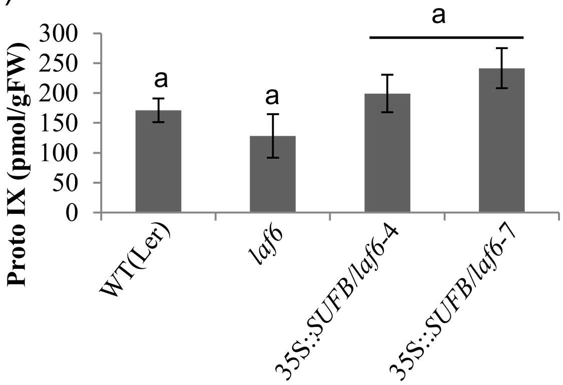

(b)

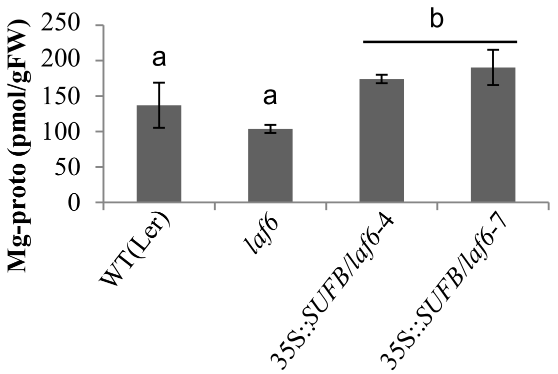

Figure S5. Proto IX (a) and Mg-proto (b) content of the developing leaves of 4-week-old laf6 and SUFB overexpressing lines in a laf6 background grown on soil under long-day conditions. Data points represent the mean ± SD of four biological replicates. Letters above each bar indicate significant differences (P < 0.05) by Tukey's multiple-comparison test.
