# Supplementary material for: The iron–sulfur cluster biosynthesis protein SUFB is required for chlorophyll synthesis, but not phytochrome signaling
Source: Plant J. 2017 Feb 8;89(6):1184–94. doi: 10.1111/tpj.13455 (PMC5347852; doi:10.1111/tpj.13455)
Supplement: Supplementary file 6 — Figure S6. Detection of protochlorophyllide a in 7‐day‐old etiolated mutant and transgenic seedlings with altered SUFB levels. [file TPJ-89-1184-s006.pdf]

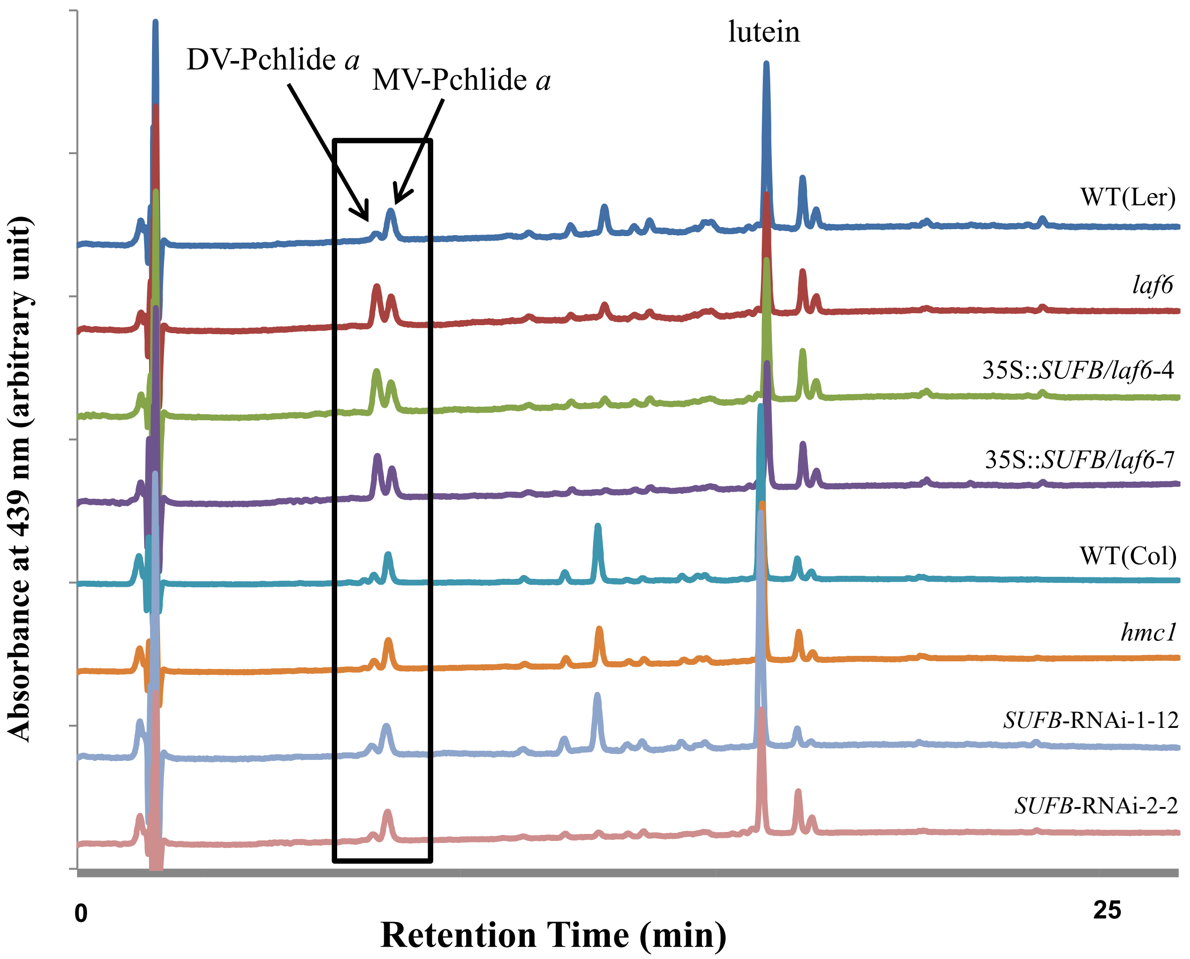

Figure S6. Detection of Pchlide a in 7-day-old etiolated mutant and transgenic seedlings with altered SUFB levels. The X axis indicates retention time. The Y axis indicates absorption at 439 nm in arbitrary units. Pchlide was detected using a diode array detector at 439 nm. The chromatograms were normalized to the height of the MV-Pchlide a peaks. DV-Pchlide a, 3,8-divinyl protochlorophylide a; MV-Pchlide a, monovinyl protochlorophylide a.
