# Supplementary material for: The iron–sulfur cluster biosynthesis protein SUFB is required for chlorophyll synthesis, but not phytochrome signaling
Source: Plant J. 2017 Feb 8;89(6):1184–94. doi: 10.1111/tpj.13455 (PMC5347852; doi:10.1111/tpj.13455)
Supplement: Supplementary file 7 — Figure S7. The effects of 0.1 mm biliverdin IXα on hypocotyl length under far‐red light of 6‐day‐old WT, laf6, hy1 and phyA seedlings. [file TPJ-89-1184-s007.pdf]

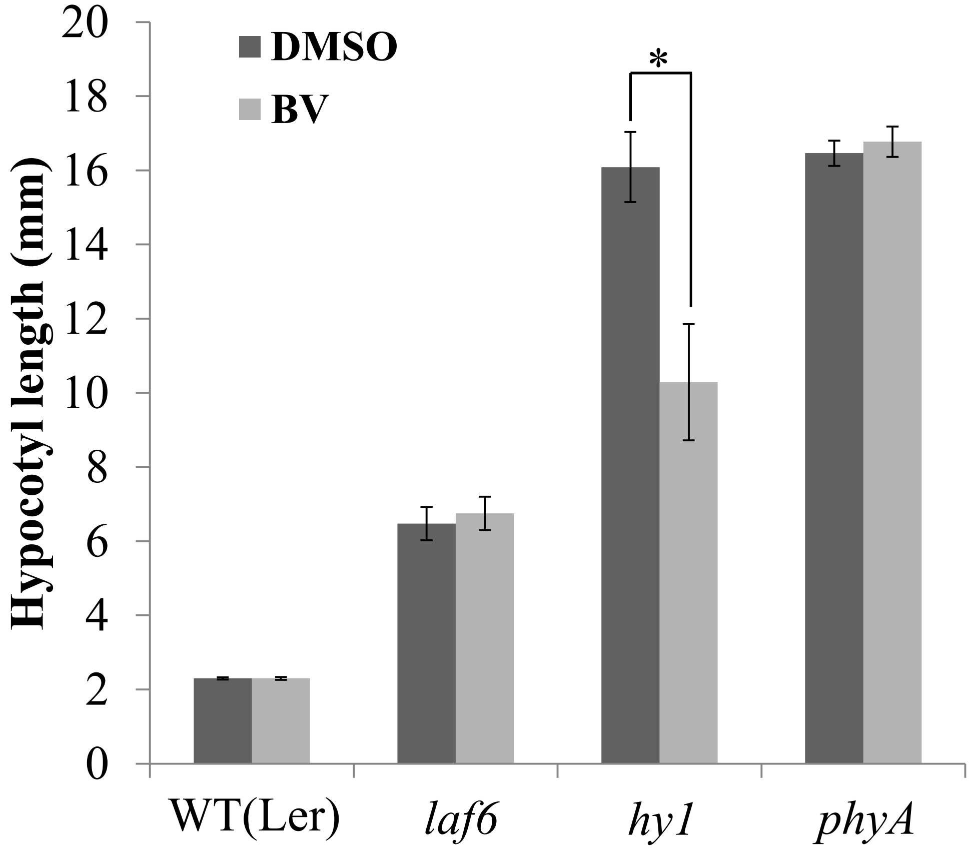

Figure S7. The effects of 0.1mM biliverdin IXα (BV) on hypocotyl length under far-red light of 6d-old WT, laf6, hy1 and phyA seedlings. DMSO was used as a control. Data shown are mean ± SE of 4 biological replicates. The asterisk (*) indicates a significant effect of BV compared to control ($P<0.05$).
