# Supplementary material for: The iron–sulfur cluster biosynthesis protein SUFB is required for chlorophyll synthesis, but not phytochrome signaling
Source: Plant J. 2017 Feb 8;89(6):1184–94. doi: 10.1111/tpj.13455 (PMC5347852; doi:10.1111/tpj.13455)
Supplement: Supplementary file 8 [file TPJ-89-1184-s008.docx]

**Supporting Information Legends**

**Figure S1.** Tetrapyrrole biosynthetic pathway. The enzymatic steps that are proposed to be affected by SUFB deficiency, 7-hydroxymethyl-chlorophyll *a* reductase (Nagane *et al*, 2010) and Mg-proto-MME cyclase (this study) are indicated. Steps known to utilize the Fe-S protein ferredoxin (Fd) are also indicated. CAO, chlorophyllide *a* oxygenase; PAO, pheophorbide *a* oxygenase.

**Figure S2.** Phenotype of 7-day-old SUFB-deficient seedlings grown on 1/2 MS medium under long-day conditions. (a) Phenotype of SUFB-deficient seedlings. (b) Chlorophyll *a* and *b* content of SUFB-deficient seedlings. Data points represent the mean ± SD of four biological replicates. Letters in black (chlorophyll *a*) or in grey (chlorophyll *b*) above each bar indicate significant differences (P < 0.05) by Tukey’s multiple-comparison test.

**Figure S3.** Analysis of chlorophyll biosynthetic intermediates in SUFB-deficient plants. (a) Proto IX and (b) Mg-proto content of the developing leaves of 4-week-old plants grown on soil under long-day conditions. Data points represent the mean ± SD of four biological replicates. Letters above each bar indicate significant differences (P < 0.05) by Tukey’s multiple-comparison test.

**Figure S4.** Complementation of *laf6* with *SUFB*. (a) Comparison of 7-day-old seedlings of *laf6* and *SUFB* overexpressing lines in a *laf6* background grown on 1/2 MS medium under long day conditions. Chlorophyll *a* and *b* content of mutant and transgenic plants. Data points represent the mean ± SD of four biological replicates. Letters in black (chlorophyll *a*) or in grey (chlorophyll *b*) above each bar indicate significant differences (P < 0.05) by Tukey’s multiple-comparison test.

**Figure S5.** Proto IX (a) and Mg-proto (b) content of the developing leaves of 4-week-old *laf6* and *SUFB* overexpressing lines in a *laf6* background grown on soil under long-day conditions. Data points represent the mean ± SD of four biological replicates. Letters above each bar indicate significant differences (P < 0.05) by Tukey’s multiple-comparison test.

**Figure S6.** Detection of Pchlide *a* in 7-day-old etiolated mutant and transgenic seedlings with altered SUFB levels. The X axis indicates retention time. The Y axis indicates absorption at 439 nm in arbitrary units. Pchlide was detected using a diode array detector at 439 nm. The chromatograms were normalized to the height of the MV-Pchlide *a* peaks. DV-Pchlide *a*, 3,8-divinyl protochlorophylide *a*; MV-Pchlide *a*, monovinyl protochlorophylide *a.*

**Figure S7.** The effects of 0.1mM biliverdin IXα (BV) on hypocotyl length under far-red light of 6d-old WT, *laf6*, *hy1* and *phyA* seedlings. DMSO was used as a control. Data shown are mean ± SE of 4 biological replicates. The asterisk (*) indicates a significant effect of BV compared to control (P<0.05).
